# Whole‐heart T_1_ mapping using a 2D fat image navigator for respiratory motion compensation

**DOI:** 10.1002/mrm.27919

**Published:** 2019-08-09

**Authors:** Giovanna Nordio, Torben Schneider, Gastao Cruz, Teresa Correia, Aurelien Bustin, Claudia Prieto, René M. Botnar, Markus Henningsson

**Affiliations:** ^1^ School of Biomedical Engineering and Imaging Sciences King's College of London London United Kingdom; ^2^ Philips Healthcare Guildford United Kingdom; ^3^ Escuela de Ingeniería Pontificia Universidad Católica de Chile Santiago Chile

**Keywords:** fat image navigator, myocardial T_1_ mapping, respiratory motion compensation

## Abstract

**Purpose:**

To combine a 3D saturation‐recovery‐based myocardial T_1_ mapping (3D SASHA) sequence with a 2D image navigator with fat excitation (fat‐iNAV) to allow 3D T_1_ maps with 100% respiratory scan efficiency and predictable scan time.

**Methods:**

Data from T_1_ phantom and 10 subjects were acquired at 1.5T. For respiratory motion compensation, a 2D fat‐iNAV was acquired before each 3D SASHA k‐space segment to correct for 2D translational motion in a beat‐to‐beat fashion. The effect of the fat‐iNAV on the 3D SASHA T1 estimation was evaluated on the T_1_ phantom. For 3 representative subjects, the proposed free‐breathing 3D SASHA with fat‐iNAV was compared to the original implementation with the diaphragmatic navigator. The 3D SASHA with fat‐iNAV was compared to the breath‐hold 2D SASHA sequence in terms of accuracy and precision.

**Results:**

In the phantom study, the Bland‐Altman plot shows that the 2D fat‐iNAVs does not affect the T_1_ quantification of the 3D SASHA acquisition (0 ± 12.5 ms). For the in vivo study, the 2D fat‐iNAV permits to estimate the respiratory motion of the heart, while allowing for 100% scan efficiency, improving the precision of the T_1_ measurement compared to non‐motion‐corrected 3D SASHA. However, the image quality achieved with the proposed 3D SASHA with fat‐iNAV is lower compared to the original implementation, with reduced delineation of the myocardial borders and papillary muscles.

**Conclusions:**

We demonstrate the feasibility to combine the 3D SASHA T_1_ mapping imaging sequence with a 2D fat‐iNAV for respiratory motion compensation, allowing 100% respiratory scan efficiency and predictable scan time.

## 1 INTRODUCTION

Cardiovascular magnetic resonance is the reference non‐invasive technique to detect and visualize different myocardial pathologies, such as amyloidosis, hypertrophic cardiomyopathies, or Fabry's disease.^1^ Late gadolinium enhancement (LGE) is widely used to image the presence of scars and focal fibrotic tissue in the myocardium. However, it cannot be used to visualize more global myocardial pathologies such as diffuse fibrosis.^2^ Quantitative parametric mapping techniques have been demonstrated to be superior for the assessment of diffuse myocardial disease (e.g., amyloidosis, Fabry's disease, or iron overload) and to quantitatively detect both local and global myocardial fibrosis.^3^ In particular, quantitative T_1_ mapping allows differentiating between healthy and diseased myocardium based on the different water content of the tissues.

Several different imaging techniques have been devised and validated for myocardial T_1_ mapping, using inversion recovery,^4^ saturation recovery,^5^ or a combination of both^6^ to perturb the longitudinal magnetization. Modified lock locker inversion recovery (MOLLI)^7^ is an inversion‐recovery based technique, which is characterized by high T_1_ precision, although it tends to underestimate the T_1_ values. Saturation recovery single‐shot acquisition (SASHA) sequence^5^ has been proposed as an alternative that uses saturation recovery pulses and is characterized by higher accuracy but lower precision compared to MOLLI. Both these T_1_ mapping sequences are typically performed as single‐shot 2D imaging techniques with a single slice being acquired per breath‐hold. A free‐breathing 3D whole‐heart technique would be preferable because it would enable complete volumetric coverage of the heart with potentially higher image resolution, allowing visualization of small myocardial structures and pathologies. A 3D SASHA^8^ imaging sequence has been recently proposed, using segmented k‐space multi‐shot acquisition rather single‐shot, which allows imaging the whole heart in free‐breathing. This technique shows similar T_1_ accuracy to 2D SASHA, but with improved precision. In the approach described in Nordio et al.,^8^ a 1D diaphragmatic navigator (1D NAV) with gating and tracking was acquired just before imaging to compensate for respiratory motion. Other techniques have been proposed for free‐breathing pre‐contrast and post‐contrast myocardial T_1_ mapping to assess diffuse fibrosis after contrast administration, which use the 1D NAV to minimize the respiratory motion.^9^, ^10^, ^11^ Although 1D NAV gating and tracking enables respiratory motion compensation for the T_1_‐weighted images, it has some limitations. The motion is estimated from the foot–head displacement of the lung–liver interface instead of the heart itself and therefore requires a motion model (typically assumed to be 0.6). In addition, the scan efficiency of the 1D NAV is typically 40–50% in healthy subjects, but can be considerably lower in patients with irregular breathing patterns. This prolonged and unpredictable scan time limits the applicability of this technique in very sick patients and precludes achieving the high spatial resolution necessary to visualize small focal fibrosis. Self‐navigation^12^ and image‐based navigator (iNAV)^13^ techniques have been recently proposed to directly estimate the respiratory‐induced motion of the heart and correct for it, enabling 100% respiratory scan efficiency and therefore shorter and predictable scan times. iNAV techniques have been used for coronary artery MRI and shown good motion estimation and correction performance.^14^, ^15^, ^16^, ^17^ However, iNAVs have not yet been extended to 3D T_1_ mapping because of the technical challenges of this technique that include: interference between the image navigator and the T_1_‐weighted images that can affect the accuracy and precision of the T_1_ measurement, low SNR of the navigators because of the effect of saturation pulses and the long T_1_ of blood, and change of contrast of the navigators during the acquisition of different T_1_‐weighted images.

In this work, we propose to address these challenges by combining the 3D SASHA imaging sequence with a fat image‐based navigator (fat‐iNAV)^18^, ^19^ to enable 2D translational motion‐corrected 3D whole‐heart T_1_ mapping with high spatial resolution in a clinically acceptable and predictable scan time. With the fat‐iNAV, epicardial fat is excited, and the navigator images are used to directly estimate the respiratory motion of the heart that has several theoretical advantages. First, with this approach the fat excitation pulses should not affect the (diagnostic) water signal and therefore not influence T_1_ quantification. Second, fat has a short T_1_ and therefore yields T_1_‐weighted navigator images with relatively high signal even for short saturation delays, whereas a water navigator would suffer from varying signal intensities for different saturation delays, potentially compromising image registration and motion estimation.

## 2 METHODS

### 2.1 Fat‐navigator acquisition and motion correction

Conventional image‐based navigator techniques excite both water and fat signals to obtain low‐resolution images of the heart at every heartbeat. Because fat magnetization recovers faster than that of water after the application of a saturation pulse, fat‐selective navigator measures a more constant signal at different saturation delays in contrast to water selective navigators, and therefore may improve motion estimation.

**Figure 1:**
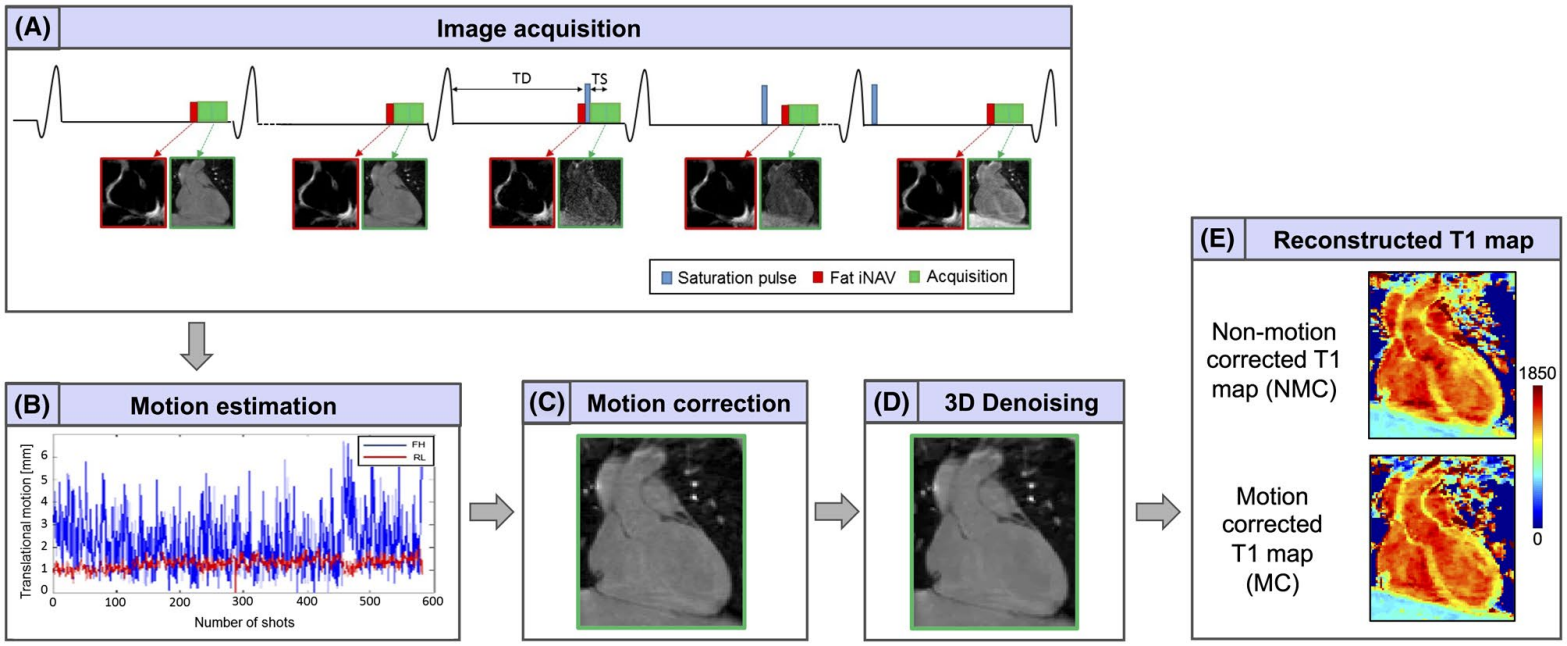
Framework of the proposed approach. (A) The 3D SASHA imaging sequence was combined with fat‐iNAVs for respiratory motion compensation. (B‐C) 2D translational motion correction was applied on the T_1_‐weighted images. (D) Before T_1_ fitting, the images were denoised using the 3D denoising algorithm proposed in Bustin et al.^21^ (E) T_1_ maps were reconstructed before and after motion correction

A schematic diagram of the framework used for image acquisition, motion estimation/correction using fat‐iNAV and T_1_ map generation is shown in Figure 1. Data were acquired using the 3D SASHA imaging sequence described in Nordio et al.^8^ A segmented 3D k‐space acquisition was used: first, all image k‐space segments with no magnetization preparation were acquired, also called infinity images, and assumed to be acquired at an infinite saturation delay. Three “pause” cardiac cycles were added between the acquisitions of these k‐space segments to ensure full recovery of the magnetization. Subsequently, an interleaved segmented acquisition was performed with preceding saturation pulse and increasing saturation delays. Fat‐iNAV was acquired immediately before the T_1_‐weighted image acquisition in mid‐diastole. Foot–head and left–right displacements of the heart were estimated from the fat‐iNAV images using a normalized cross‐correlation registration algorithm and then used to correct the k‐space of each T_1_‐weighted image using the phase shift property of the Fourier transform.

The acquisition parameters used for the fat‐iNAV acquisition were: 3 × 3 mm^2^ in plane image resolution, FOV = 300 × 300 mm^2^, slice thickness = 20 mm, 15° flip angle, TR/TE = 1.5 ms/7.2 ms, fat selective = 1331 binomial pulse,^20^ SENSE factor of 2, orientation in coronal view, linear profile order, Cartesian fast echo planar imaging acquisition with 5 excitations and an EPI factor of 9, resulting in a temporal resolution of 73 ms. Translational motion correction and T_1_ mapping reconstruction were performed offline using MATLAB (MathWorks, Natick, MA). SENSE reconstruction was performed for both non‐motion‐corrected and motion‐corrected 3D SASHA data sets. A denoising method was applied to the T_1_‐weighted images of both the 2D and 3D SASHA acquisitions before parametric fitting to reduce the noise and improve both image quality and T_1_ precision of the T_1_ maps.^21^, ^22^ A 3‐parameter fitting model was then used to reconstruct the T_1_ maps offline for the 2 data sets with a pixel‐wise fitting approach.

### 2.2 Phantom experiment

A phantom with 9 agar/NiCl_2_ vials with T_1_ values ranging from 250–1500 ms was scanned using the proposed fat‐iNAV T_1_ mapping approach.^23^ The T_1_ phantom data was acquired with an inversion recovery spin‐echo (IRSE) acquisition, providing reference T_1_ values, and the 2D and 3D SASHA imaging sequences with fat‐iNAV for comparison purposes. Sequence parameters for IRSE were: FOV = 150 × 150 mm^2^, spatial resolution = 1.95 × 1.95 mm^2^, 10 mm slice thickness, TR between inversion pulses = 7000 ms, TE  =  6 ms, 20 inversion times varying from 50–5000 ms. The imaging parameters for the 2D SASHA sequence were: FOV = 300 × 280 mm^2^, spatial resolution = 1.7 × 2.1 × 10 mm^3^, flip angle = 70°, TR/TE = 2.4/1.2, SENSE factor of 2. The imaging parameters for the 3D SASHA technique were: FOV = 300 × 300 mm^2^, spatial resolution = 2 × 2 × 4 mm^3^, reconstructed image resolution = 2 × 2 × 2 mm^3^, flip angle = 35°, TR/TE = 3.2/1.6, SENSE factor of 2, balanced SSFP (bSSFP) acquisition, Cartesian trajectory with radial k‐space shutter, low‐high profile order, and coronal view. For the 3D SASHA, a total of 8 images were acquired, and 3 pause heart cycles were performed between the acquisitions of k‐space segments of the infinity image.

To investigate potential interference between the fat‐iNAV and 3D SASHA acquisition, 2 different slices of the 3D SASHA acquisition were analyzed, 1 in the same slice location as the fat‐iNAV (slice with fat‐iNAV) and 1 parallel to the iNAV at a more distal location (16 mm) (slice without fat‐iNAV). T_1_ measurements were then obtained in the 2 slices and compared with the Bland‐Altman analysis and the Spearman test for correlation.

To compare the navigator signal between the fat‐iNAV and conventional non‐spectrally selective navigator excitation, the T_1_ phantom and a phantom containing fat were scanned using the 3D SASHA imaging sequence with either of the 2 navigator acquisition approaches.^14^ The signal of the fat‐iNAV was measured in the phantom containing fat, whereas the signal of the conventional navigator was measured in the vial of the T_1_ phantom with a T_1_ value similar to that of blood.

### 2.3 In vivo experiments

The study was performed in accordance with the Declaration of Helsinki (2000). All subjects recruited in this study provided written informed consent with study approval from the Institutional Review Board (1/11/12). Ten healthy subjects (age = 31 ± 3 y, 6 male) were imaged on a 1.5T Ingenia Philips MR system (Philips Healthcare, Best, the Netherlands) with a 28‐channel coil array and using the 3D SASHA imaging sequence with the fat‐iNAV described in Figure 1. For comparison, 2D SASHA was acquired during breath‐hold in a mid‐ventricular slice, with the same imaging parameters used for the phantom experiment. The imaging parameters used for the 3D SASHA were FOV = 300 × 300 × 86–106 mm^2^, spatial resolution = 2 × 2 × 4 mm^3^, reconstructed image resolution = 2 × 2 × 2 mm^3^, flip angle = 35˚, TR/TE = 3.2/1.6, SENSE factor of 2, bSSFP acquisition, Cartesian acquisition with radial k‐space shutter, low‐high profile order and coronal orientation. The 2D SASHA imaging sequence was acquired in short axis view, whereas the 3D SASHA and fat iNAV were acquired in a coronal orientation to mitigate through plane motion and facilitate retrospective in‐plane motion correction. Whole‐heart coverage in combination with coronal orientation and 4 mm slice thickness required the acquisition of a larger number of slices compared to the previously published short‐axis 3D SASHA that only covered the left‐ventricle and used 8‐mm slice thickness.

For 1 of the subjects, the 3D SASHA acquisition was performed twice, both using the fat‐iNAV and the conventional non‐spectrally selective excitation navigator, to compare the respiratory motion of the heart estimated using the 2 different navigators. In both cases, a normalized cross‐correlation registration algorithm was used for motion estimation.

For 2 representative subjects, the proposed 3D SASHA imaging technique with the fat‐iNAV was compared with 3D SASHA using the diaphragmatic navigator with tracking only to evaluate the performance of the 2 different navigator techniques for motion estimation and correction.

For 3 representative subjects, the proposed 3D SASHA imaging technique with the fat‐iNAV was also compared with the original implementation of the 3D SASHA with 1D NAV, gating, and tracking, to compare the image quality and quantitative values of the motion‐corrected T_1_ maps for both techniques.

### 2.4 Image analysis

T_1_ maps were reconstructed using a 3‐parameter fitting approach, both before and after translational motion correction. For the phantom study, a ROI was manually drawn in the 9 vials and the mean and SD of the T_1_ measurements were measured as an estimate of the accuracy and the precision of the T_1_ map, respectively. For statistical analysis, GraphPad Prism v5 for Windows (GraphPad Software, La Jolla, CA) was used. The T_1_ values measured in the phantom in the 2 different slices (with and without fat‐iNAV) were compared using a Bland‐Altman plot and the correlation was calculated using a Spearman test. The mean percent difference was calculated to compare the T_1_ values estimated by the reference IRSE and the 2D and 3D SASHA sequences. For the in vivo study, a ROI was manually drawn in the myocardial septum and the mean and the SD of the T_1_ values were measured as an estimate of, respectively, the accuracy and precision. A Kruskal‐Wallis test was used to compare the accuracy and precision measured on the 2D and 3D SASHA T_1_ maps before and after motion correction. The in vivo data set, acquired in coronal view, were reformatted in the short axis view to the same slice as 2D SASHA for comparison using Horos software v1.1.7. An AHA segmentation was used to compare the accuracy and precision of the 3D SASHA with fat‐iNAV before and after motion correction and for a 3D visualization of the left ventricle. The cardiac volume was represented in 16 segments and 3 slices (apex, mid, and base), with the segment 17 corresponding to the blood pool. The value in each segment was averaged within the 10 healthy subjects.

## 3 RESULTS

### 3.1 Phantom experiment

Supporting Information Table S1 shows the mean and SD measured on the 9 vials of the T_1_ phantom using the IRSE, 2D SASHA, and 3D SASHA imaging sequences. For the 3D SASHA sequence, the T_1_ values were measured in 2 different slices (slice with and without fat‐iNAV). There was a good agreement between the IRSE and the 3D SASHA imaging sequence, for both slices. The correlation between the T_1_ measurements for the 2 different 3D SASHA slices is shown in Supporting Information Figure S1, where a significant correlation (r^2^ = 0.99, *P* < 0.0001) was found. Supporting Information Figure S2 shows the Bland‐Altman plot comparing the T_1_ values measured in the 2 slices of the 3D SASHA acquisition. There is no bias between the 2 set of measurements, while the 95% limits of agreements are equal to 12.5 ms. Supporting Information Figure S3 shows the T_1_ maps of the 2D SASHA and 3D SASHA imaging sequences and the mean percent difference between the T_1_ values estimated by the reference IRSE and the 2D and 3D SASHA imaging sequences.

Supporting Information Figure S4 shows the signal of the fat (red) image navigator and the water (blue) image navigator (acquired with a non‐spectrally selective excitation pulse), measured at different saturation times. The signal for the fat‐iNAVs is similar for all saturation times, whereas it increases significantly for the water image navigator with increasing saturation delay time and particularly for the infinity image water iNAV.

### 3.2 In vivo experiments

Supporting Information Figure S5 shows the motion‐corrected T_1_‐weighted images and T_1_ maps acquired with the 3D SASHA imaging sequence using fat‐iNAV or 1D diaphragmatic navigator with tracking (scaling factor 0.6, no gating) for 2 representative subjects. The fat‐iNAV resulted in improved delineation of the myocardium and higher quality T_1_ maps suggesting improved motion estimation and correction with fat‐iNAV compared to the 1D NAV.

**Figure 2:**
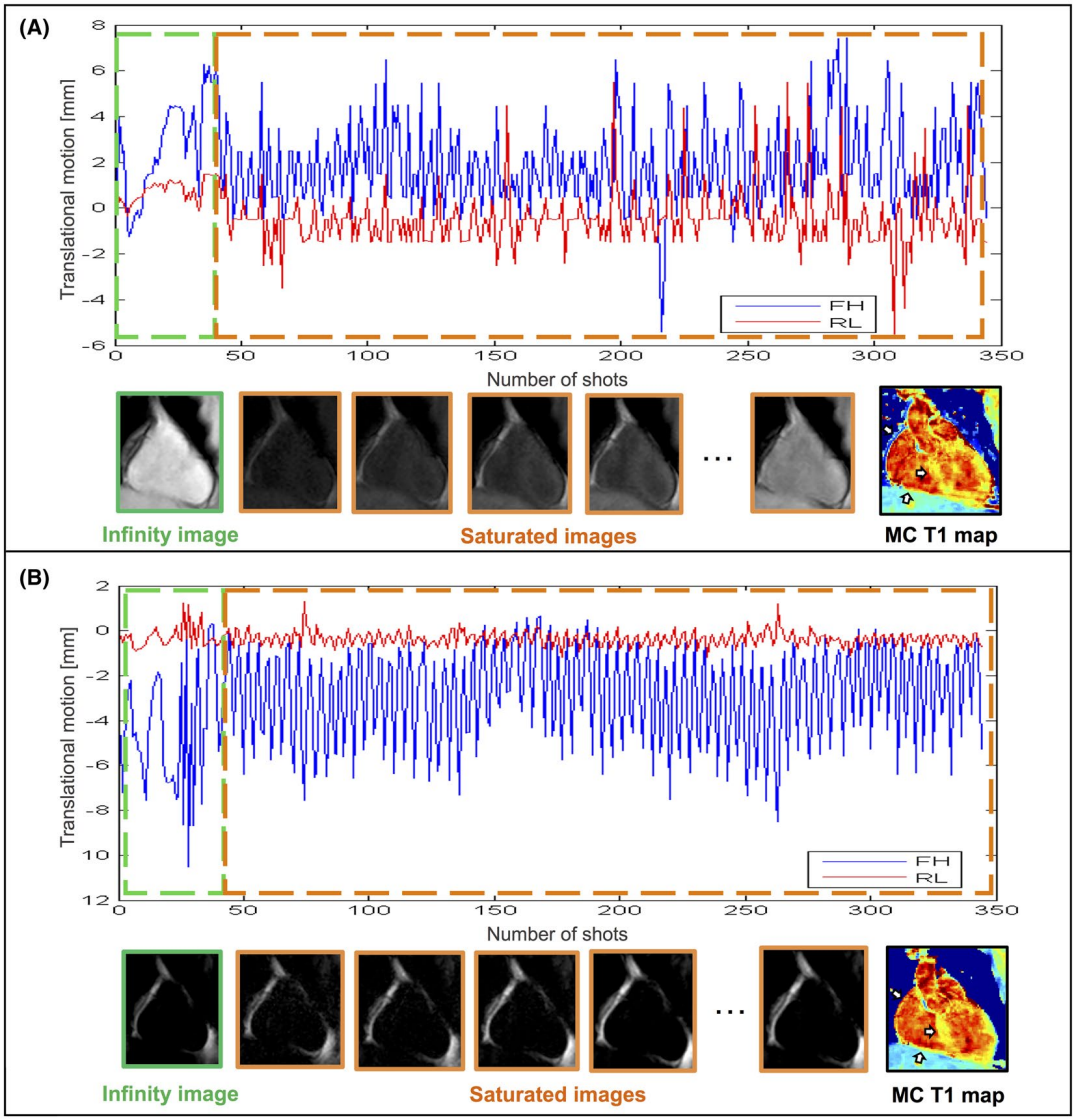
Foot–head (FH blue signal) and right–left displacement (RL red signal) of the heart measured with the conventional navigator (A) and the proposed fat‐iNAV (B) for 1 representative subject. The corresponding image navigators of the non‐saturated images (infinity image, green), and of the saturated images (orange) are shown below the displacements, together with the motion‐corrected (MC) 3D SASHA T_1_ maps, respectively, using the motion estimated from the water (A) and the fat (B) iNAVs

Figure 2 compares the translational motion estimated using a conventional non‐spectrally selective excitation for the water image navigator (Figure 2A) and the fat‐iNAV (Figure 2B) for 1 healthy subject. The change in contrast of the acquired 3D SASHA images with different T_1_‐weighting are reflected in the navigators with non‐spectrally selective excitation that causes a change in the trend of the translational motion estimated between the acquisition of the infinity images (green box) and the beginning of the acquisition of the saturated images (orange box). In addition, the change in contrast of the water image navigator makes it challenging to correct to a common reference iNAV used for the image registration, which causes errors in the motion estimation. Consequently, residual motion also affects the quality of the reconstructed T_1_ map, where the precision is lower compared to the T_1_ map reconstructed using the fat‐iNAV, as shown in Figure 2.

**Figure 3:**
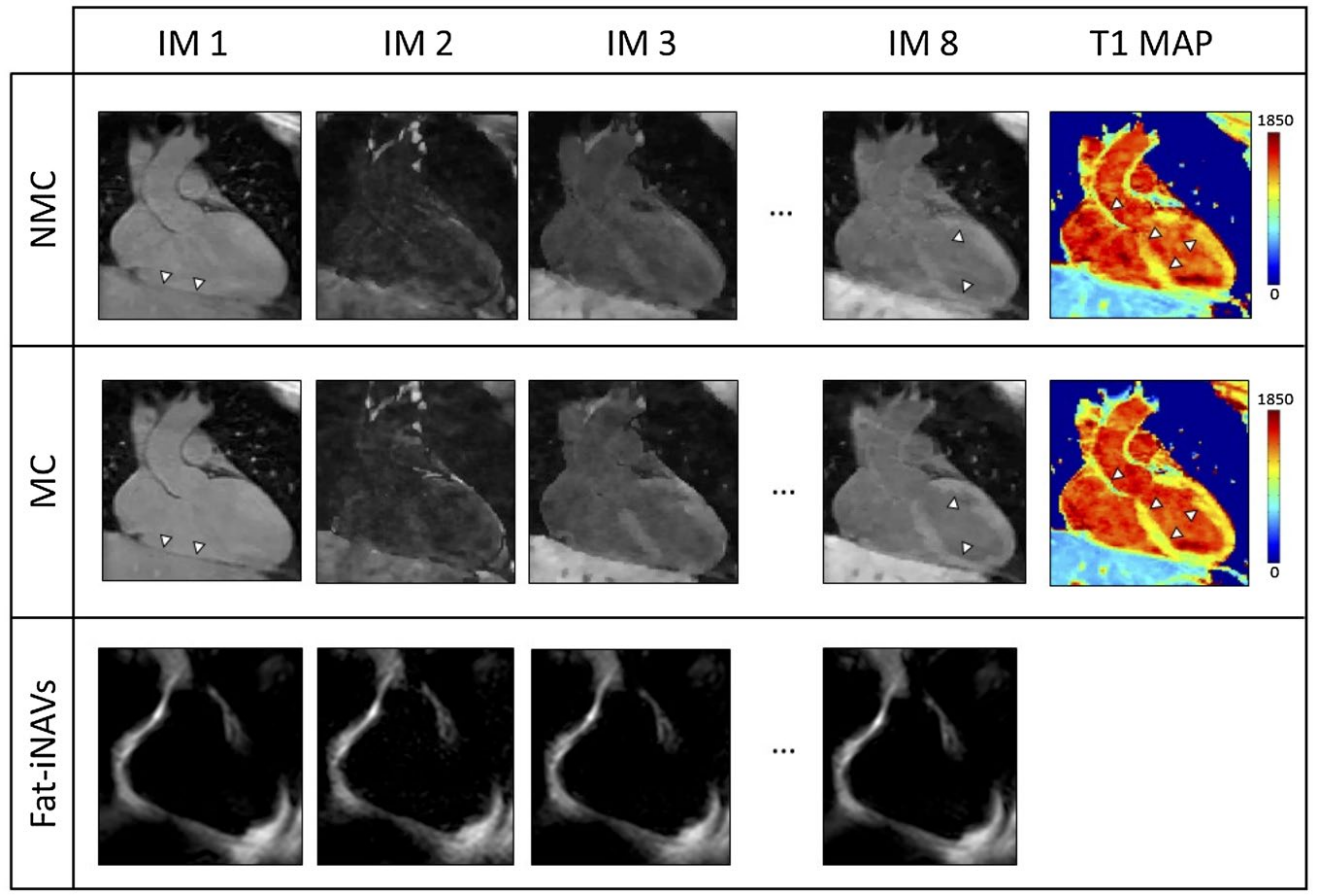
T_1_‐weighted images and myocardial T_1_ map non‐motion‐corrected (NMC) and motion‐corrected (MC) for 1 representative subject. In the bottom row the fat‐iNAVs of the corresponding T_1_‐weighted images are shown

**Figure 4:**
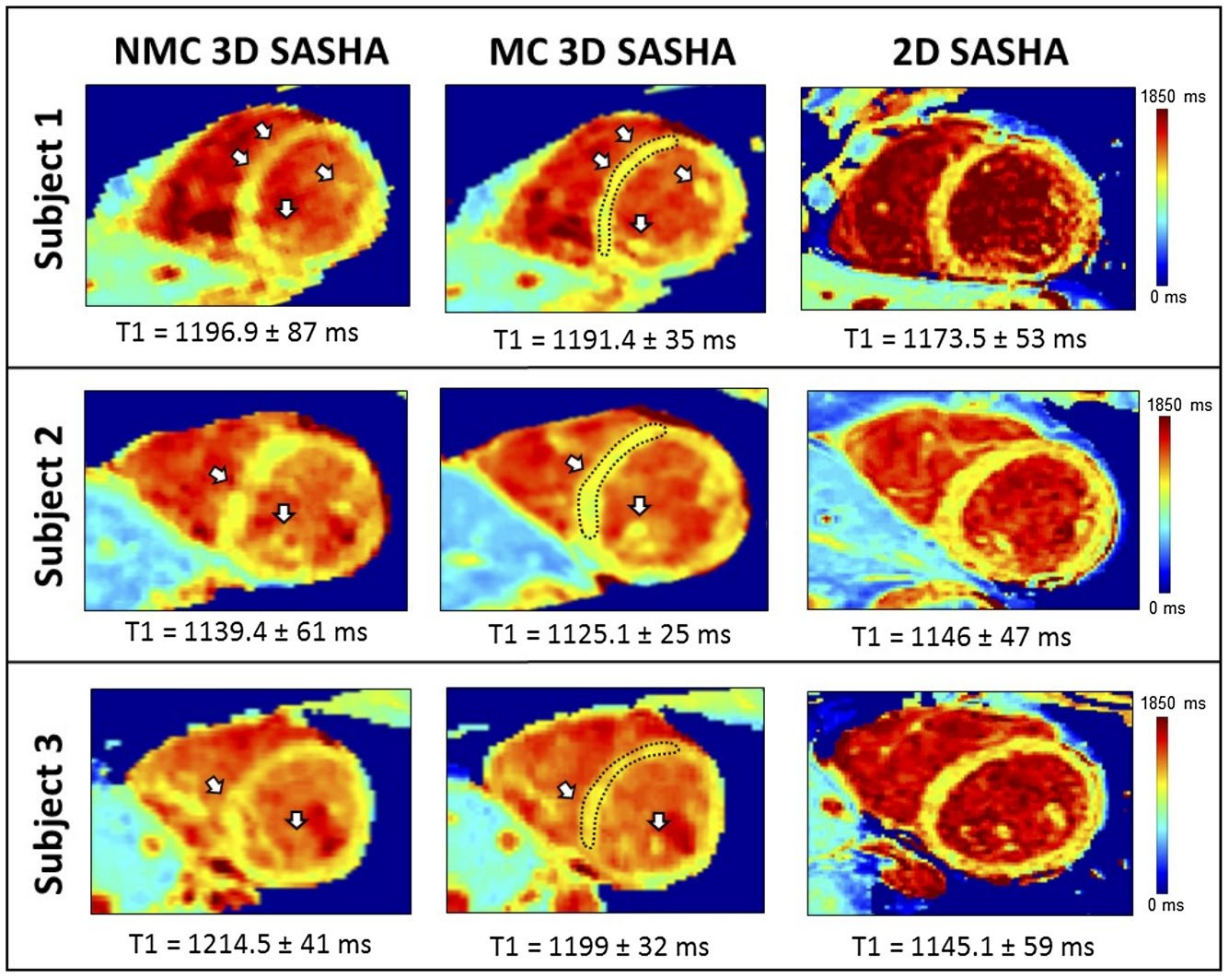
Short axis view of the myocardial T_1_ maps using the non‐motion‐corrected (NMC) and motion‐corrected (MC) free‐breathing fat‐iNAV 3D SASHA and breath hold 2D SASHA imaging sequences for 3 representative subjects. Mean and SDs were measured in the myocardial septum

**Figure 5:**
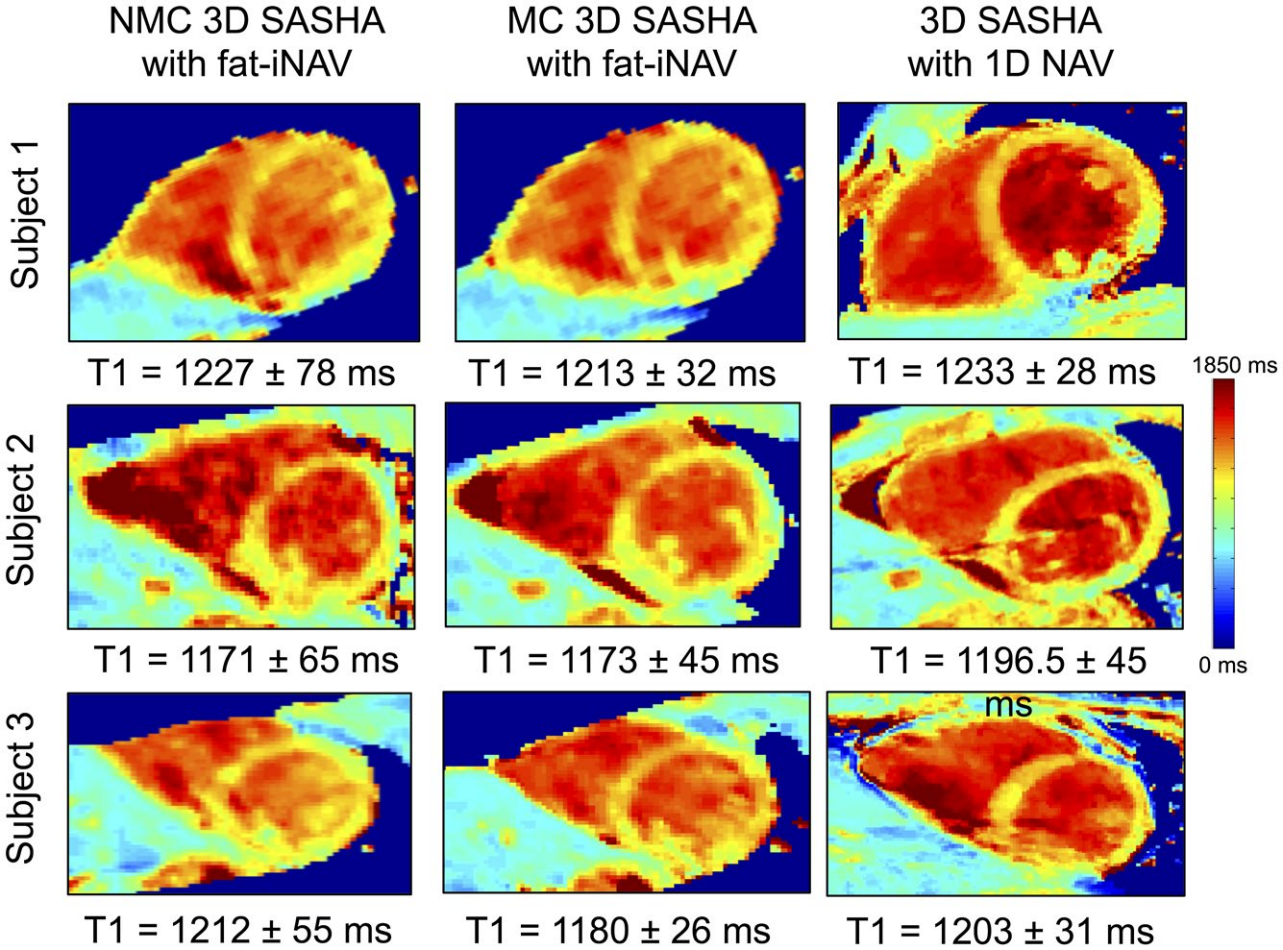
Short axis view of the myocardial T_1_ maps using the non‐motion‐corrected (NMC) and motion‐corrected (MC) free‐breathing fat‐iNAV 3D SASHA and 3D SASHA with 1D diaphragmatic navigator imaging sequences, with both gating and tracking, for 3 representative subjects. Mean and SDs were measured in the myocardial septum

Figure 3 shows the T_1_‐weighted images and the 3D SASHA myocardial T_1_ map for 1 representative subject with no motion correction (NMC) and after 2D translational motion correction (MC). There was an improvement in the delineation of the myocardium and papillary muscle after motion correction both in the T_1_‐weighted images and the T_1_ map. The corresponding fat‐iNAVs are shown for the different saturation times with no visible change in image contrast. Figure 4 shows the reformat of the 3D SASHA T_1_ maps in short axis view for 3 representative subjects before and after respiratory motion correction, in comparison with the corresponding 2D SASHA T_1_ maps. After translational motion correction the delineation of the myocardial borders and the papillary muscles improves, as shown in Figure 4. There is good agreement between the T_1_ measured in the septum of the myocardium between the 3D SASHA and 2D SASHA T1 maps, with an improvement in the precision for the 3D SASHA T_1_ map, because of the denoising method^21^ and because of the higher SNR compared to the 2D SASHA. The precision of the 3D SASHA T_1_ maps further improves after translational motion correction as shown in Supporting Information Figure S6 for all the 10 subjects. This is also confirmed by the AHA plot (Supporting Information Figure S7), which shows that the precision is improved for most segments after motion correction, with the exception of the inferior segments. The average total scan time for the 3D SASHA imaging sequence was 7 ± 0.8 min. Figure 5 shows the T_1_ maps acquired with the proposed 3D SASHA with fat‐INAV, before and after motion correction, and with the original implementation of the 3D SASHA with 1D NAV for gating and tracking. After motion correction, the image quality achieved with the proposed 3D SASHA with fat‐iNAV is lower compared to the original implementation, with reduced delineation of the myocardial borders and papillary muscles.

## 4 DISCUSSION

In this study, we propose a new fat image‐based navigator for free‐breathing 3D myocardial T_1_ mapping. The fat‐iNAV has been combined with the previously proposed 3D SASHA myocardial T_1_ mapping technique,^8^ enabling increased coverage, high spatial resolution, 100% respiratory scan efficiency, and predictable scan time. Ideally, the fat‐iNAV only excites signal from epicardial fat, without affecting the water signal of the 3D SASHA T_1_ mapping acquisition and therefore does not introduce systematic bias of the T_1_ measurement. This has been demonstrated through the T_1_ phantom experiment, which shows good agreement between the T_1_ values measured in 2 different slices, 1 without fat‐iNAV and 1 corresponding with the fat‐iNAV (Supporting Information Figure S1).

The binomial fat excitation pulse of the iNAV should in theory only affect the fat signal. However, in practice some saturation of the water signal was observed, as shown in Supporting Information Figure S8, particularly in the blood pool. We hypothesize that through‐plane flow during the spatial–spectral binomial pulses can lead to saturation artifacts. In the case of through‐plane flow, the blood only experiences a subset of the spatially selective RF pulses that are spaced a few milliseconds apart, leading to flow‐dependent saturation. A shorter binomial RF pulse train with fewer subpulses could be used to potentially mitigate this effect at the expense of narrowing the spectral stopbands that would increase sensitivity to B_0_ inhomogeneity.^24^ However, the flow‐related saturation should only affect the blood signal and not the myocardium (and therefore myocardial T_1_ values) that experiences minimal motion in the through‐plane (anterior–posterior) direction during the binomial saturation pulse. In patients with significant amounts of epicardial fat, the image navigator will have more tissue from which to estimate respiratory motion, which may yield better respiratory motion correction. In the current work, fat suppression was used to minimize signal from subcutaneous and pericardial fat in the T_1_ maps, primarily to avoid motion artifacts arising from these tissues. Visualization of fat is of interest to detect fatty infiltration of the myocardium. Although fat‐iNAV RF‐pulses may cause saturation effects of the fat signal for the T_1_ maps, the use of low flip angles combined with few RF excitations and the short T_1_ of fat means more than 85% of the longitudinal magnetization remains immediately after the fat‐iNAV has been performed. Therefore, there is only a minor loss in fat SNR in the regions where the fat‐iNAV and T_1_ map overlap.

The signal of the fat‐iNAVs is relatively constant between the acquisitions of the different saturation time points, as shown in Supporting Information Figure S2. This is also confirmed in vivo, where the contrast of the fat‐iNAVs is visually maintained constant along the whole acquisition, because of a faster recovery of the fat signal after the saturation pulse compared to the water signal, ensuring a robust estimation of the respiratory motion of the heart (Figure 2). Non‐spectrally selective excitation results in different contrast at different saturation times and this can affect the estimation of the respiratory motion, as shown in Figure 2A. The incorrect estimation of the translational motion using the water iNAVs has a direct impact on the motion‐corrected T_1_ map, where residual motion affects the image quality and the delineation of the myocardium. In addition, the precision is lower on the T_1_ map motion‐corrected using the water iNAVs compared to the T_1_ map motion‐corrected using the fat‐iNAVs. The 2D fat‐iNAV also showed a better detection, and consequently motion correction, compared to the 1D diaphragmatic navigator and this results in improved myocardium delineation on the T_1_ maps as well as improved T_1_ accuracy and precision (Supporting Information Figure S5). Additional experiments are needed to measure the accuracy of motion estimation of the 2 techniques. However, the 3D SASHA T_1_ maps with fat‐iNAV and 100% scan efficiency (no gating) yielded reduced image quality compared with the 3D SASHA T1 maps using a 1D diaphragmatic navigator with both gating and tracking (Figure 5). To obtain similar image quality with the fat‐iNAV as with the gated NAV scans, more advanced motion correction techniques such as affine or non‐rigid correction will be required, which would better approximate the respiratory motion of the heart.^25^, ^26^, ^27^ Alternatively, more motion tolerant k‐space trajectories could be used for T_1_ mapping such as radial acquisitions.^28^, ^29^, ^30^ This could also permit discarding a small subset of the most motion corrupted k‐space data without introducing detrimental undersampling artifacts.

Compared to the original implementation of the 3D SASHA imaging technique, where a 1D diaphragmatic NAV was used for gating and tracking, the acquisition has been considerably accelerated using the fat‐iNAV reducing the scan time from 12 to 7 min. Because of the reduced scan time, it was possible to achieve higher image resolution, increasing the slice resolution from 8 to 4 mm compared to the original implementation of 3D SASHA with 1D NAV, and to reconstruct images with isotropic resolution, which permits to retrospectively reformat the images in an arbitrary plane. T_1_ values measured in the myocardial septum using the 3D SASHA technique are comparable to that of the 2D SASHA, with an improvement in the precision after respiratory motion correction (Figure 4 and Supporting Information Figure S6). In addition, the SNR of the 3D SASHA acquisition is higher than that of the 2D SASHA, and this contributes as well to the improvement of the precision of the T_1_ map. The precision of the 3D SASHA T_1_ maps further improves after translational motion correction, as indicated by the AHA plot in the Supporting Information Figure S7. However, in the inferior regions, the precision is still lower after motion correction, likely because of residual motion. Again, the use of more advanced motion compensation techniques and motion tolerant k‐space trajectories could reduce the residual motion and further improve the precision in these segments.^27^, ^31^, ^32^, ^33^

Parallel imaging has been used to accelerate the acquisition, however more advanced undersampling techniques such as compressed sensing^34^ or low‐rank^35^ may achieve even higher acceleration factors and consequently may further improve image resolution.^36^

## 5 CONCLUSION

In this study, we proposed a new approach for respiratory motion correction for 3D free‐breathing myocardial T_1_ mapping, using a fat image‐based navigator. The proposed fat‐iNAV allows for 100% respiratory scan efficiency and predictable scan time, as well as high spatial resolution.

## CONFLICT OF INTEREST

Torben Schneider works for Philips Healthcare.

## Supporting information


**FIGURE S1** Correlation between the T_1_ values measured on the T_1_ phantom in the 2 different slices of the 3D SASHA acquisition, 1 corresponding to the fat‐iNAV (slice with fat‐iNAV) and 1 outside the area of excitation of the fat‐iNAV (slice without fat‐iNAV). The identity line is also indicated in the graph
**FIGURE S2** Bland‐Altman plot comparing the 3D SASHA acquired on the 2 different slices (without and with fat‐iNAV). The mean and the 95% limits of agreement are reported in the graph
**FIGURE S3** (A) T_1_ maps of the T_1_ phantom using the 2D and 3D SASHA sequences. (B) Mean percent difference between the T_1_ values estimated by the reference IRSE and the 2D (in blue) and 3D SASHA (in red) sequences
**FIGURE S4** Comparison of the signal measured in the conventional (blue) and fat (red) image navigator, for each different T_1_‐weighted image of the T_1_ phantom
**FIGURE S5** T_1_‐weighted images and T_1_ maps of 2 representative subjects, acquired with the proposed 3D SASHA sequence with the fat‐iNAV and with the 3D SASHA sequence with the 1D diaphragmatic navigator with tracking only. The fat‐iNAV allows to improve myocardium delineation (white arrows), as well as to improve T_1_ accuracy and precision
**FIGURE S6** Accuracy and precision measured on the non‐motion‐corrected (NMC, blue), motion‐corrected (MC, red) 3D SASHA, and 2D SASHA (yellow) T_1_ maps for the 10 healthy subjects. The T_1_ values were measured in the septum of the myocardium
**FIGURE S7** AHA plot of the left ventricle, shown for the non‐motion‐corrected (NMC) and motion‐corrected (MC) 3D SASHA (n = 10 subjects). The cardiac volume is represented in 16 segments and 3 slices (apex, mid, and base), whereas the center represents the blood pool. The precision improves after motion correction, although low precision was found in the inferior wall after motion correction because of residual motion artefacts
**FIGURE S8** Transversal view of the T_1_‐weighted images and the reconstructed 3D SASHA T_1_ map of 3 subjects. The position of the fat‐iNAV is indicated by the pink box. Saturation effect of the blood pool are visible particularly in subjects 2 and 3
**TABLE S1** Mean and SDs measured in the 9 vials of the T_1_ phantom using the inversion recovery spin‐echo (IRSE), 2D SASHA, and 3D SASHA for 2 different slices
