# Supplementary material for: Whole‐heart T1 mapping using a 2D fat image navigator for respiratory motion compensation
Source: Magn Reson Med. 2019 Aug 9;83(1):178–87. doi: 10.1002/mrm.27919 (PMC6791811; doi:10.1002/mrm.27919)

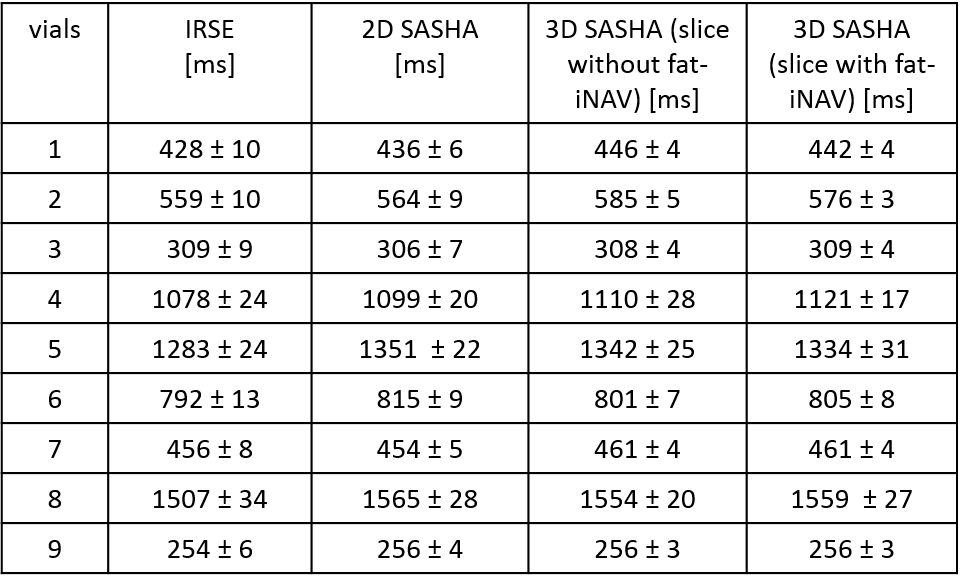
Supporting Information Table S1:

Supporting Information Figure S1:


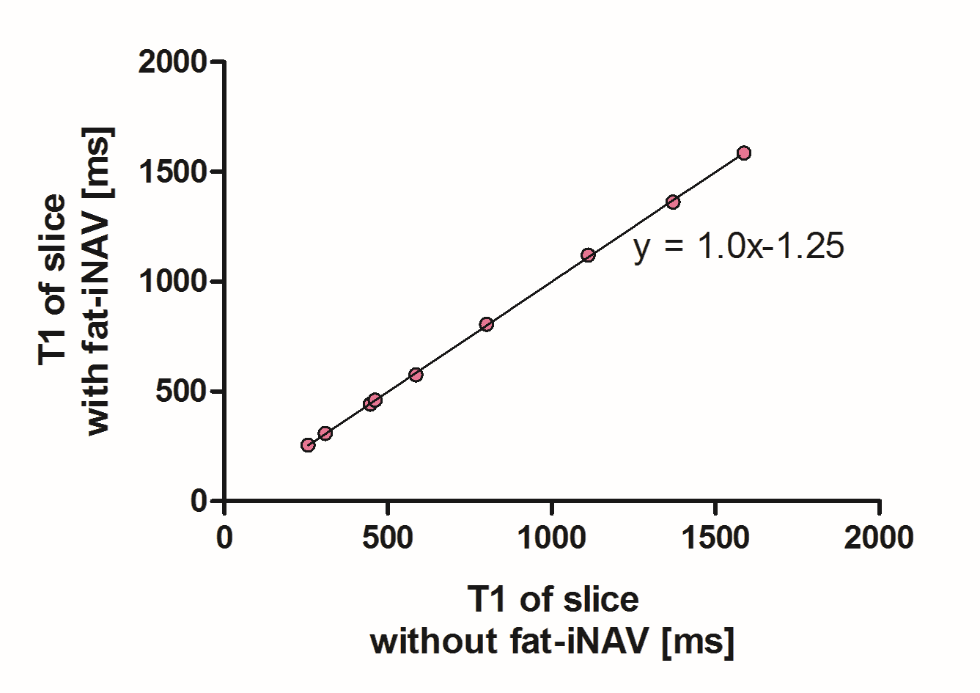


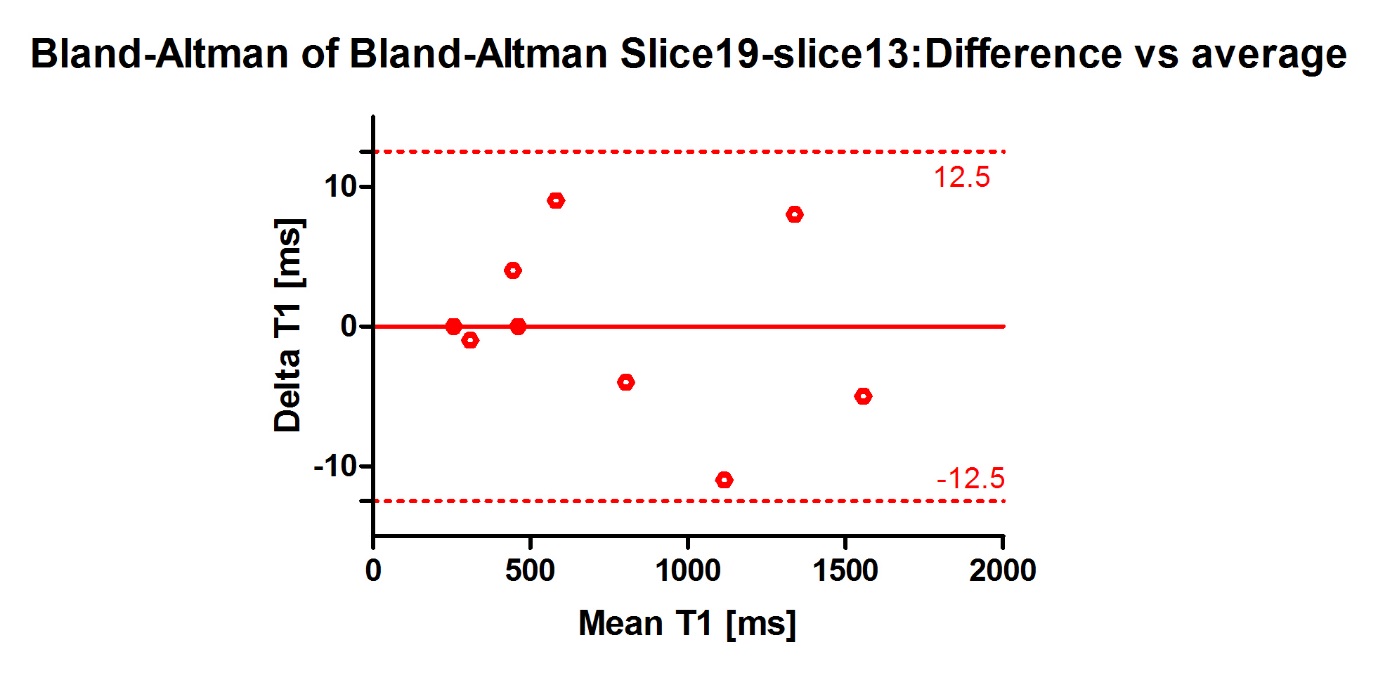
Supporting Information Figure S2:


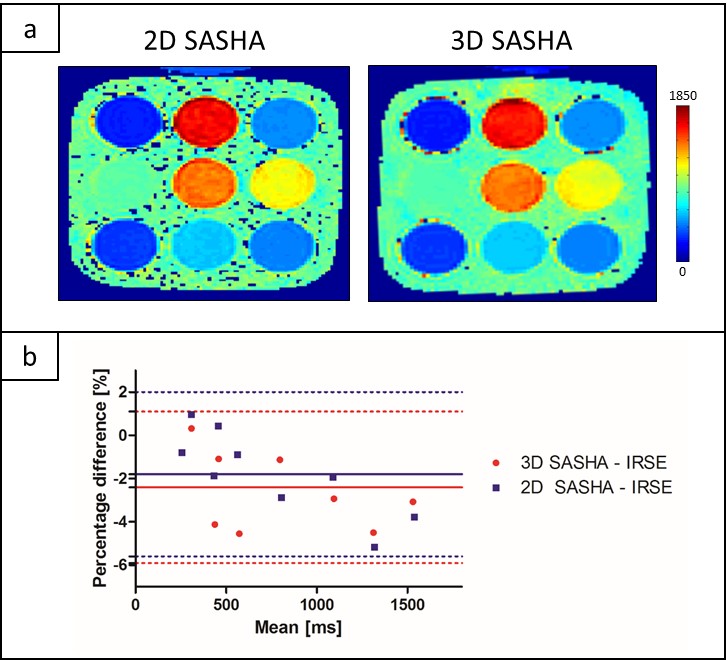
Supporting Information Figure S3:

Supporting Information Figure S4:


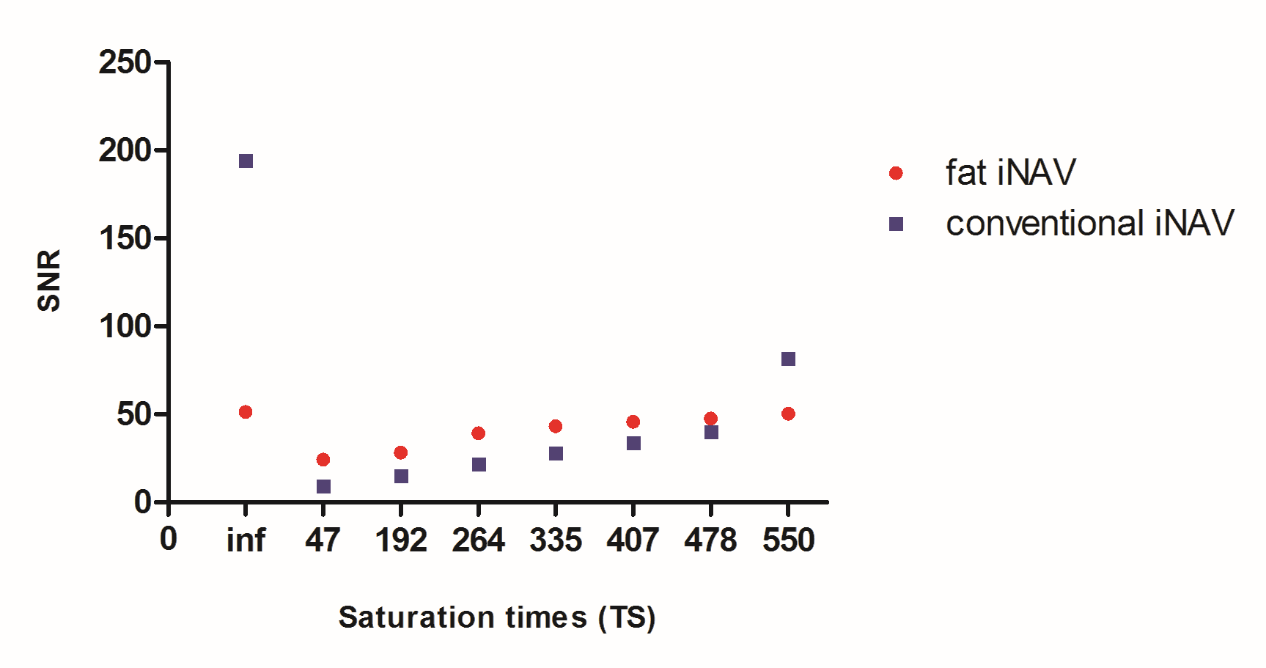


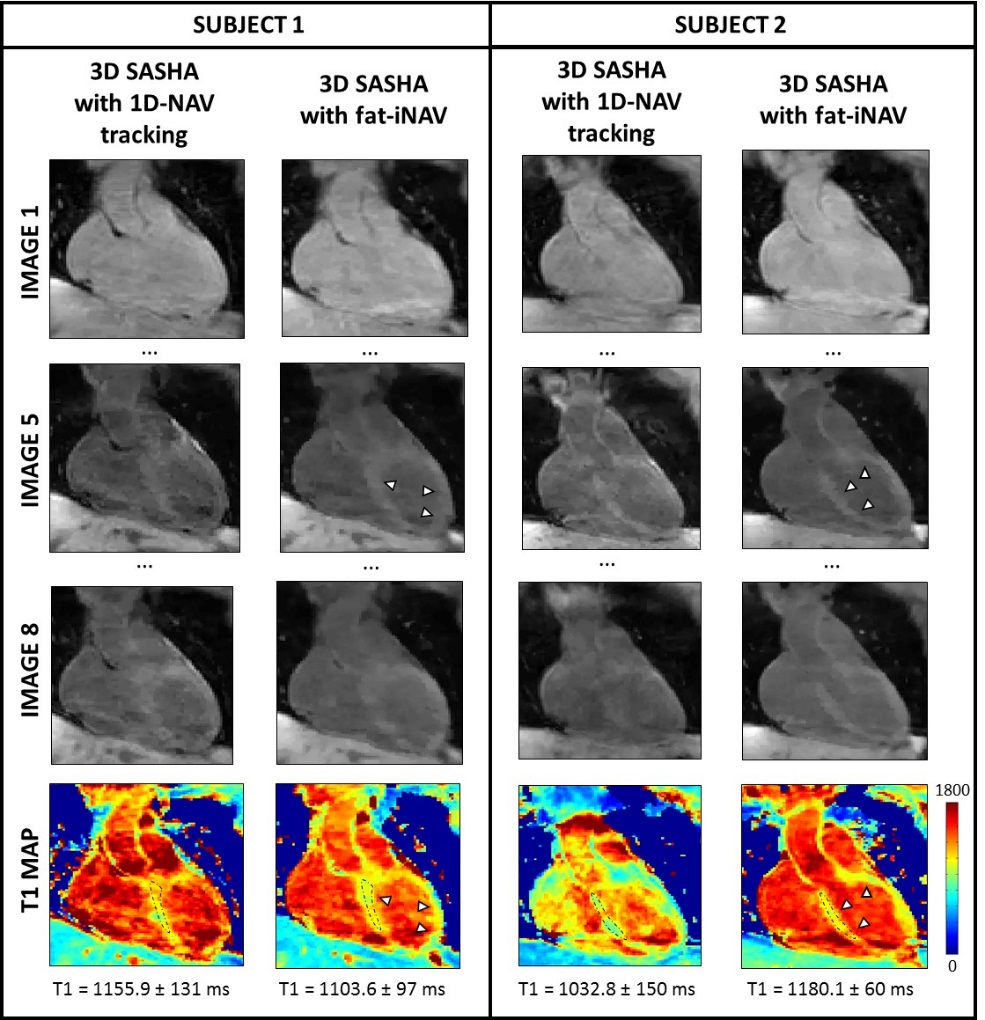
Supporting Information Figure S5:


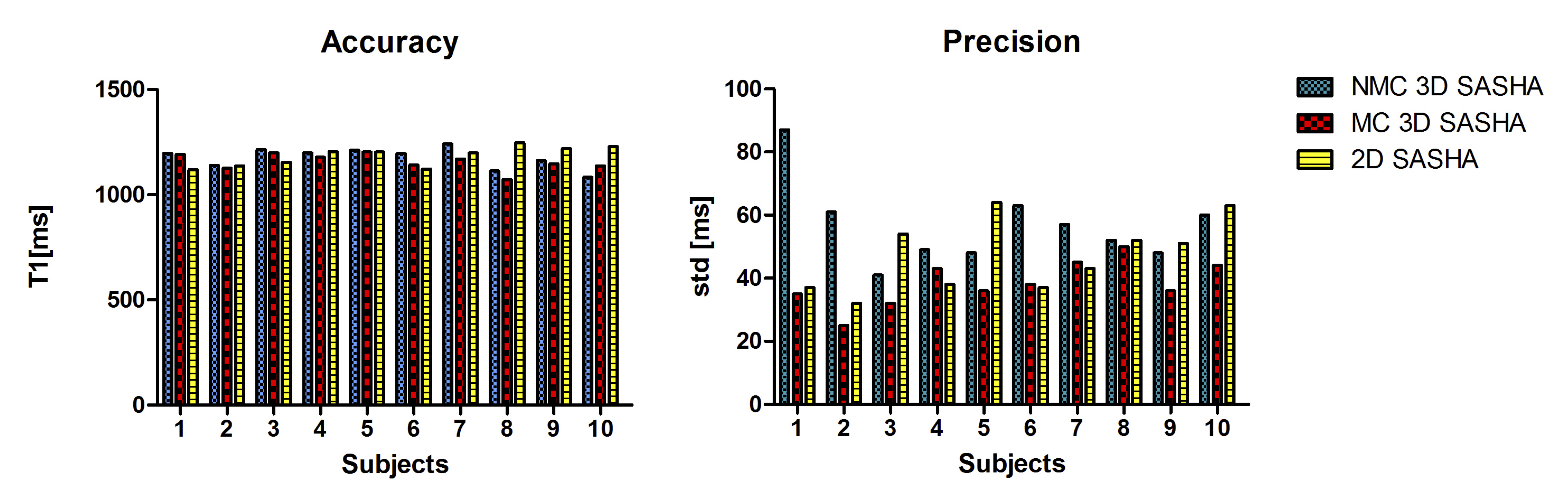
Supporting Information Figure S6

Supporting Information Figure S7:


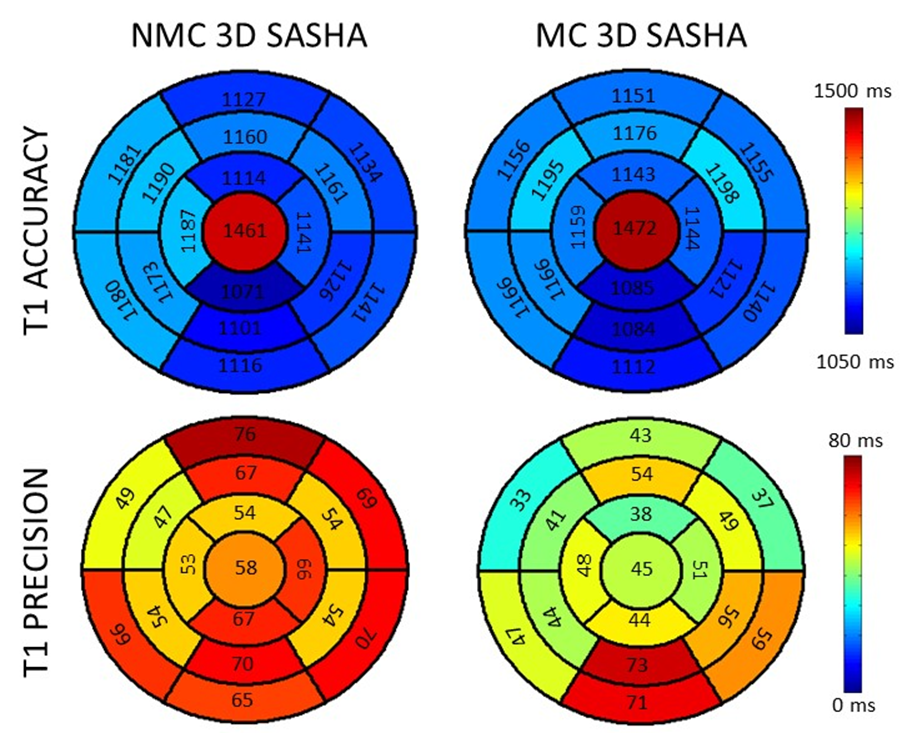


Supporting Information Figure S8:


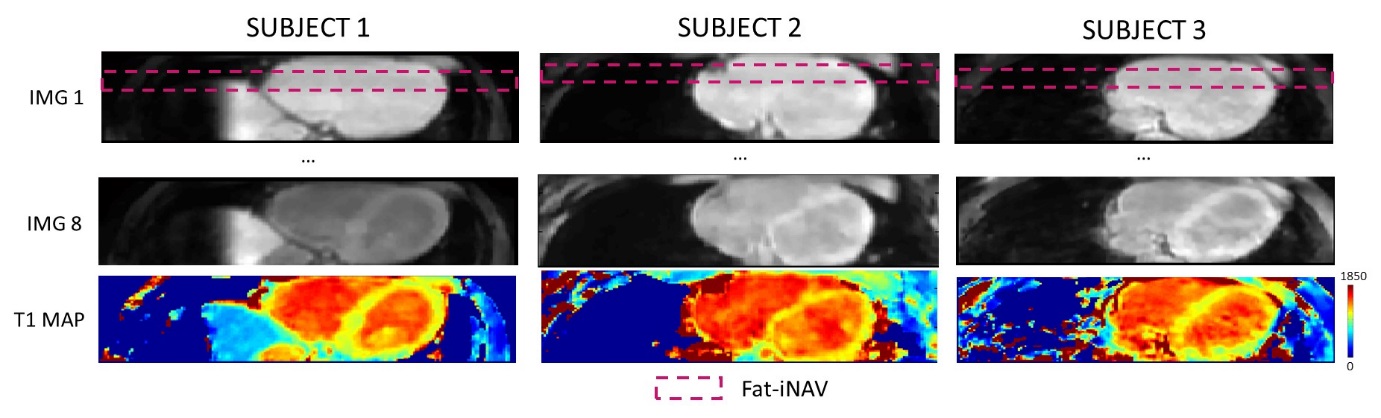

Supplement: Supplementary file 1 — FIGURE S1 Correlation between the T1 values measured on the T1 phantom in the 2 different slices of the 3D SASHA acquisition, 1 corresponding to the fat‐iNAV (slice with fat‐iNAV) and 1 outside the area of excitation of the fat‐iNAV (slice without fat‐iNAV). The identity line is also indicated in the graph FIGURE S2 Bland‐Altman plot comparing the 3D SASHA acquired on the 2 different slices (without and with fat‐iNAV). The mean and the 95% limits of agreement are reported in the graph FIGURE S3 (A) T1 maps of the T1 phantom using the 2D and 3D SASHA sequences. (B) Mean percent difference between the T1 values estimated by the reference IRSE and the 2D (in blue) and 3D SASHA (in red) sequences FIGURE S4 Comparison of the signal measured in the conventional (blue) and fat (red) image navigator, for each different T1‐weighted image of the T1 phantom FIGURE S5 T1‐weighted images and T1 maps of 2 representative subjects, acquired with the proposed 3D SASHA sequence with the fat‐iNAV and with the 3D SASHA sequence with the 1D diaphragmatic navigator with tracking only. The fat‐iNAV allows to improve myocardium delineation (white arrows), as well as to improve T1 accuracy and precision FIGURE S6 Accuracy and precision measured on the non‐motion‐corrected (NMC, blue), motion‐corrected (MC, red) 3D SASHA, and 2D SASHA (yellow) T1 maps for the 10 healthy subjects. The T1 values were measured in the septum of the myocardium FIGURE S7 AHA plot of the left ventricle, shown for the non‐motion‐corrected (NMC) and motion‐corrected (MC) 3D SASHA (n = 10 subjects). The cardiac volume is represented in 16 segments and 3 slices (apex, mid, and base), whereas the center represents the blood pool. The precision improves after motion correction, although low precision was found in the inferior wall after motion correction because of residual motion artefacts FIGURE S8 Transversal view of the T1‐weighted images and the reconstructed 3D SASHA T1 map of 3 subjects. The position of the fat‐iN [file MRM-83-178-s001.docx]
